# Fibrosis in systemic sclerosis: common and unique pathobiology

**DOI:** 10.1186/1755-1536-5-S1-S18

**Published:** 2012-06-06

**Authors:** Swati Bhattacharyya, Jun Wei, Warren G Tourtellotte, Monique Hinchcliff, Cara G Gottardi, John Varga

**Affiliations:** 1 Departments of Medicine and Pathology, Feinberg School of Medicine, Northwestern University, Chicago, IL, USA

## Abstract

Fibrosis in systemic sclerosis (SSc), a complex polygenic disease associated with autoimmunity and proliferative/obliterative vasculopathy, shares pathobiologic features in common with other fibrosing illnesses, but also has distinguishing characteristics. Fibroblast activation induced by transforming growth factor-β (TGF-β), Wnts and innate immune receptors, along with oxidative stress and reactive oxygen species (ROS) are implicated in pathogenesis. On the other hand, the roles of endothelial-mesenchymal differentiation and bone marrow-derived fibrocytes remain to be established. Fibrotic responses are modulated by transcriptional activators and cofactors, epigenetic factors, and microRNAs that can amplify or inhibit ligand-induced signaling. The nuclear orphan receptor PPAR-γ appears to be important in governing the duration and intensity of fibroblast activation and mesenchymal progenitor cell differentiation, and defects in PPAR-γ expression or function in SSc may underlie the uncontrolled progression of fibrosis. Identifying the perturbations in signaling pathways and cellular differentiation programs responsible for tissue damage and fibrosis in SSc allows their selective targeting using novel compounds, or by innovative uses of already-approved drugs (drug repurposing).

## Introduction

Systemic sclerosis (SSc) is serious chronic fibrosing disease with high mortality without any effective therapy. Progressive fibrosis in the lungs, heart, kidneys and other organ leads to their dysfunction and eventual failure. The inability to identify appropriate patients for treatment with existing or novel targeted disease-modifying therapies is due to multiple factors: 1) complex nature of SSc, with concomitant vascular injury, autoimmunity, inflammation and fibrosis; 2) lack of bona fide animal models of disease; 3) poorly understood genetic and environmental risk factors; and 4) significant patient-to-patient clinical heterogeneity in terms of disease course and outcomes. This overview focuses on the pathobiological features that SSc shares with other fibrosing conditions, and some that are unique to SSc.

### Fibroblast activation in SSc

Fibroblasts explanted from the lesional skin of SSc patients synthesize increased amounts of collagen and fibronectin in vitro [1,2]. Moreover, SSc fibroblasts show constitutive production of cytokines and chemokines; and spontaneous myofibroblast transdifferentiation [3]. Whether these phenotypic features reflect cell-autonomous perturbations of intracellular signaling molecules and pathways due to genetic or epigenetic alterations, or reflect paracrine/autocrine fibroblast activation triggered by extracellular cues [4,5] (Table 1 and 2) remains unanswered.

**Table 1:** Intracellular signal mediators showing aberrant expression in SSc

Molecule	Increased expression/activity	Decreased expression/activity
Egr-1, Egr-2	⇑	
Sp1	⇑	
p300/CBP	⇑	
Fli-1		⇓
Smad7		⇓
Ski/Sno		⇓
Nab2		⇓
PTEN		⇓
PPAR-gamma		⇓
microRNA29		⇓

**Table 2:** Extrinsic mediators of fibroblast activity potentially implicated in SSc

Cytokines
TGF-β
IL-4
IL-13
IL-17
IL-33

Growth factors, peptides and bioactive lipids

Wnt family (Wnt3a, Wnt10b, others)
CTGF (matricellular protein)
PDGF
IGFBP-5
Endothelin-1
Adenosine
Lysophosphatidic acid (LPA)
Prostaglandin F

Chemokines

CXCL12
MCP-1

Autoantibodies

Antibody to Topo I
Anti-fibroblast antibody
Anti-PDGF antibody

### Cell types and cell fate switching in fibrosis

The fibroblast is the proximal effector cell directly responsible for fibrosis. However, recent studies indicate that bone marrow-derived mesenchymal progenitors such as fibrocytes and monocytes might traffic to damaged tissue and undergo in situ differentiation into activated fibroblasts and myofibroblasts. Non-fibroblastic cell lineages (such as epithelial or endothelial cells or adipocytes) can also differentiate into fibroblasts and myofibroblasts via processes involving Notch, Snail, Slug and S100A4 (FSP-1). Hypoxia, TGF-β and Wnts promote the transition of precursor and non-fibroblastic cell types toward an activated myofibroblast phenotype, whereas PPAR-γ promotes the maintenance of cellular quiescence. To date, no convincing evidence has emerged to implicate cellular plasticity in the pathogenesis of fibrosis in SSc.

### Persistent fibrosis: innate immune recognition signaling via TLRs

**Figure 1:**
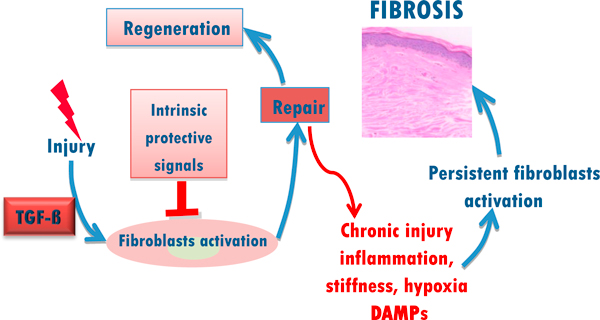
**Vicious cycle of FIBROSIS**. Innate immune signaling alters self-limited repair into sustained fibrogenic process. Following injury, fibroblasts undergo a regulated activation. Once repair has been accomplished, tissue regeneration is complete. Prolonged injury leads to damage, causes activation of toll like receptors and sustained fibroblast activation culminating in excessive fibrogenesis.

Innate immune recognition signaling via toll-like receptors (TLRs) appears to play an important role in the persistent fibrotic response in SSc. The ability to recognize pathogen-associated molecular patterns via pattern recognition receptors (PRRs) is a critical aspect of the host ability to respond to foreign microorganisms [6]. We have demonstrated that TLR3 and TLR4 are expressed in normal fibroblasts and transduce signals from lipopolysaccharide (LPS) as well as endogenous TLR ligands, the so-called damage-associated molecular pattern (DAMPs) such as polyinosinic:polycytidylic acid and matrix components such as hyaluronic acid, and fibronectin-EDA (Fn-EDA) [7,8]. In the liver, TLR4 activated by LPS plays a critical role in fibrosis, with suppression of BAMBI and sensitization to TGF-β as the underlying mechanisms [9]. We speculate that in SSc tissue injury, exacerbated by hypoxia and accumulation of reactive oxygen species (ROS), up-regulate fibroblast TLR4 expression and/or activity on mesenchymal stromal cells, contributing to TLR-mediated amplification of TGF-β signaling. Moreover, oxidative damage and tissue remodeling generating DAMPs such as low molecular weight hyaluronan degradation products, Fn-EDA, Tenascin C, and biglycan; cellular stress proteins such as HMGB1 and Hsp60; and nucleic acids and immune complexes, each of which might activate fibroblasts via TLRs engagement. In this way, the accumulation of tissue damage-associated endogenous TLR ligands serve as a danger signals that perpetuate fibroblasts activation. We speculate that DAMP-induced TLR signaling might serve as the critical "switch" that, when turned on, converts a self-limited tissue repair process into one of persistent and unchecked fibroblasts activation resulting in progressive fibrosis in SSc. This concept is illustrated in Figure 1.

### Fibroblast activation

Transforming growth factor-β is the pre-eminent signal for connective tissue synthesis, and is considered as a "core pathway" in both normal wound healing and pathological fibrosis. Microarray-based analysis of genome-wide changes in gene expression has shown that a subset of SSc patients with aggressive disease show evidence of activation of TGF-β-dependent signaling pathways in the lesional skin [10-12].

### The non-receptor tyrosine kinase c-Abelson (c-Abl) and Egr-1

In normal fibroblasts, TGF-β induces Smad-independent activation of c-Abl, a Src family non-receptor tyrosine kinase implicated in chronic myelogenous leukemia (CML) [13-15]. Endogenous c-Abl is required for the profibrotic responses induced by TGF-β in vitro. Imatinib, a small molecule inhibitor of c-Abl kinase used for the treatment of CML and gastrointestinal stromal tumors, blocked the stimulation of collagen synthesis, fibroblast proliferation and myofibroblast transdifferentiation elicited by TGF-β. Moreover, imatinib suppressed the constitutively elevated collagen gene expression in SSc fibroblasts (Hinchcliff et al, manuscript submitted). In vivo, imatinib attenuated the severity of lung and skin fibrosis induced by bleomycin in mice [13,15]. It is noteworthy that imatinib blocks signaling via receptor for PDGF. An important downstream target of c-Abl appears to be Egr-1, a prototypical member of zinc finger transcription factors. Egr-1 expression is typically induced at sites of injury by cytokines, lipids and mechanical injury. Egr-1 is implicated in cell proliferation, differentiation and survival [16]. TGF-β was shown to induce Egr-1 expression in normal dermal fibroblasts [17,18]. Fibroblasts lacking Egr-1 showed loss of collagen stimulation in response to TGF-β, identifying Egr-1 as a novel downstream mediator of profibrotic TGF-β responses. Moreover, Egr-1 induces the production of TGF-β and TGF-β receptors, and also stimulates the coactivator and histone acetyl transferase p300, thereby greatly amplifying TGF-β-induced cellular responses [17] and Ghosh AK et al, MS submitted). Expression of the oxidase enzyme Nox4, responsible for ROS generation in TGF-β-stimulated fibroblasts, is directly stimulated by Egr-1[19]. Lesional skin biopsies from SSc patients shows increased Egr-1 expression and activity. Furthermore, mice lacking Egr-1 have markedly attenuated fibrotic response to bleomycin in vivo [20]. Egr-1 thus emerges potent fibrogenic mediator in the pathogenesis of SSc.

### Aberrant activation of the developmental Wnt-β-catenin program in SSc

The Wnts constitute a large family of secreted signaling glycoproteins with key roles in embryonic development and organogenesis. In contrast to embryogenesis, where the Wnt pathway is active, in adults Wnt signaling is normally tightly regulated. Canonical Wnt signaling is initiated by ligand binding to Frizzled (FZD) and Low-density lipoprotein receptor-related protein (LRP) surface receptors, stabilizing cytosolic β-catenin. In the absence of ligand, β-catenin is phosphorylated by glycogen synthetase kinase 3-β (GSK3-β), leading to its ubiquitination and proteasomal degradation [21]. In Wnt-stimulated fibroblasts, the FZD receptor inhibits GSK-3β activity and blocks β-catenin degradation. β-catenin consequently translocates into the nucleus where is regulates target gene transcription via TCF/LEF binding. Expression of many Wnt target genes is regulated in a cell type-specific manner [22,23]. Genomewide profiling reveals elevated Wnt/β-catenin signature in patients with pulmonary fibrosis [24-26] and SSc [11,27]. These observations are further confirmed by detection of constitutive GSK-3β phosphorylation and nuclear β-catenin localization in fibrotic lesion [25,26,28] We found constitutive Wnt-β-catenin activation in the lungs of patients with SSc-associated pulmonary fibrosis [29] and in lesional skin from dcSSc [30]. The source of Wnt ligand, and the triggers for aberrant Wnt signaling are currently unknown. In light of the ability of canonical Wnt pathway to stimulate fibroblast activation and mesenchymal progenitor cell differentiation [28,30], and the association of transgenic Wnt10b expression with scleroderma-like skin fibrosis and subcutaneous lipoatrophy in the mouse [31], hyperactivation of canonical Wnt signaling in SSc skin biopsies, while suppressing adipogenesis, aberrant Wnt signaling is likely to be important in the pathogenesis of SSc, and is an interesting potential target for therapy.

### Bioactive lipids

Certain prostanoids inhibit fibrotic responses and tissue remodeling through a variety of mechanisms [32], whereas prostaglandin F (PGF_2α_) is elevated in IPF, and stimulates collagen production and fibroblast proliferation [33]. Lysophosphatidic acid (LPA), generated from membrane phospholipids via hydrolysis, induces fibroblast chemotaxis and CTGF production [34]. Mice with targeted deletion of LPA1 are protected from bleomycin-induced lung fibrosis. A recent study indicates that LPA induces avβ6-mediated TGF-β activation in epithelial cells, contributing to sustained autocrine TGF-β signaling [35]. LPA might be a potential therapeutic target for fibrosis of the lungs, kidneys and liver, and the availability of small molecule LPA1 makes this approach particularly appealing.

### Peroxisome proliferator-activated receptor-γ: intrinsic negative regulation of fibroblast activation and differentiation

The nuclear orphan receptor peroxisome proliferator-activated receptor gamma (PPAR-γ) modulates TGF-β signaling and mesenchymal cell plasticity. Naturally occurring PPAR-γ ligands including prostanoids such as 15d-prostaglandin J_2 _(15d-PGJ_2_) drive nuclear PPAR-γ accumulation and DNA binding. Rosiglitazone and other members of the thiazolidinedione class of insulin-sensitizing drugs are synthetic PPAR-γ agonists used in type 2 diabetes. PPAR-γ is involved in energy metabolism, adipogenesis, bone and vascular biology and immune responses, and its abnormalities have been linked to lipodystrophy, atherosclerosis, pulmonary hypertension, cancer, obesity and inflammation.

Recent studies have revealed an entirely novel function for PPAR-γ in connective tissue homeostasis and matrix remodeling as a cell-intrinsic anti-fibrotic pathway. In normal fibroblasts, activation with either natural (15d-PGJ_2_) or synthetic (rosiglitazone) PPAR-γ ligands resulted in abrogation of TGF-β-induced collagen production and Smad3-dependent transcriptional responses [36]. Subsequent studies showed that PPAR-γ blocked histone acetyl transferase p300 recruitment due to competition for limiting amounts of this indispensable Smad3 coactivator (squelching), resulting in inhibition of Smad-dependent transcriptional responses [37,38]. Moreover, PPAR-γ blocks the activation function of Egr-1 [39]. Incubation of TGF-β-stimulated alveolar epithelial cells with PPAR-γ ligands prevented epithelial to mesenchymal transition (EMT) and the associated suppression of E-cadherin levels [40]. PPAR-γ plays a fundamental role in regulating mesenchymal cell lineage fate determination and can shift progenitor cell differentiation along fibrogenic or non-fibrogenic pathways.

### Impaired PPAR-γ in fibrosis and SSc: role in pathogenesis

Mouse studies have demonstrate that fibroblast-specific gene targeting of PPAR-γ resulted in exaggerated skin fibrosis in bleomycin-treated mice [41], and PPAR-γ deletion targeted to follicular stem cells was associated with scarring alopecia [42]. On the other hand, rosiglitazone attenuated bleomycin-induced dermal fibrosis via induction of PPAR-γ signaling [39]. Reduced PPAR-γ expression or function is associated with various forms of fibrosis in vivo which may account for the predictable association of fibrosis with subcutaneous and visceral lipoatrophy. Remarkably, the process of aging itself is associated with declining PPAR-γ expression [43].

PPAR-γ expression and activity are impaired in the lesional skin in SSc [44]. Furthermore, PPAR-γ expression is inversely correlated with TGF-β signaling. Aberrant expression or activity of profibrotic cues such as TGF-β and Wnts might account for reduced PPAR-γ function, which in turn contributes to the progression of fibrosis [45]. Together, these studies findings implicate defective expression and function of PPAR-γ in SSc, and raise the possibility that activating PPAR-γ using ligand agonists such as the thiazolidenediones, novel non-agonist ligands, or the triterpenoid CDDO, might be novel therapeutic approaches.

## Conclusion

Cell-intrinsic alterations in SSc fibroblasts including deregulated TGF-β and Wnt signaling, altered expression or function of c-Abl and Egr-1; persistent TLR activation by DAMPs, hypoxia and mechanical forces and a functional deficiency of endogenous repressors of fibroblast differentiation and collagen production such as PPAR-γ and microRNAs, contribute to persistent biosynthetic and mechanical activity, and progressive fibrosis [46]. The roles of progenitor cell differentiation and cellular transitions in the development of SSc fibrosis and the molecular factors regulatory processes remain to be defined in SSc. The entire repertoire of molecular pathways contributing to fibroblasts activation and progressive fibroproliferation needs to be annotated. Emerging cellular and molecular targets provide a plethora of appealing opportunities for therapy, as well as for the discovery and validation of pathogenesis-based biomarkers for clinical studies. We believe that these advances presage rapid research progress toward improved outcome for management of patients with SSc.

## Competing interests

The authors declare that they have no competing interests.
